# Victorian healthcare experience survey 2016–2018; evaluation of interventions to improve the patient experience

**DOI:** 10.1186/s12913-021-06336-0

**Published:** 2021-04-07

**Authors:** Eunice Wong, Felix Mavondo, Lidia Horvat, Louise McKinlay, Jane Fisher

**Affiliations:** 1 BehaviourWorks Australia, Monash Sustainable Development Institute, Monash University, Melbourne, PO Box 8000, Monash University LPO, Clayton, VIC 3800 Australia; 2 School of Public Health and Preventive Medicine, Monash University, Melbourne, Australia; 3 Department of Marketing, Monash University, Melbourne, Australia; 4 Safer Care Victoria, Department of Health, Victoria, Australia

**Keywords:** Patient experience survey, Patient experience, Public hospital, Interventions, Australia

## Abstract

**Background:**

Patient experience is recognised as a quality of care indicator and increasingly health services are working on achieving set targets and improving their performance. Interventions at the point of care targeting communication with patients, patient engagement in care processes and discharge planning are associated with better patient experience. However, their efficacy and application to different contexts are still unclear. The aims were to describe the interventions implemented by health services to improve patient experience, their impact on overall patient experiences and specific experiences in areas of communication, discharge planning, patient education on treatment/tests, the physical environment and access to care.

**Methods:**

Secondary data analysis of the Victorian Healthcare Experience inpatient surveys reported in September 2016 and 2018 and content analysis of interventions published in the Victorian Quality Account for 2017 from 59 public health services in Victoria, Australia. The interventions were categorised using an adapted taxonomy of professional interventions by the Cochrane EPOC Review Group. Univariate tests and confirmatory factor analysis were conducted to test measure invariance across the 2016 and 2018 groups and examine the association between each of the intervention categories on overall patient experience measure and specific outcome measures.

**Results:**

This study found that the overall patient experience was consistent (93%) between 2016 and 2018 samples. In comparing impact, a single intervention rather than none or multiple interventions in communication, respect and dignity and treatment and disease education areas were associated with a higher level of the overall patient experience. Interventions in waiting time, access to service, care continuity and emotional support categories were associated with a decrease in overall patient experience.

**Conclusion:**

This study found that to improve the overall patient experience, more focus is needed on evidence-based interventions in dignity and respect and emotional support. Furthermore, the choice of interventions should be guided by evidence of their efficacy and prioritising implementing one intervention well, provides more gains.

**Supplementary Information:**

The online version contains supplementary material available at 10.1186/s12913-021-06336-0.

## Background

Patient experience is commonly defined as the range of interactions that patients have with the health care system, their care from health plans, and doctors, nurses, and staff in hospitals, physician practices and other health care facilities [1–3]. Consistent positive associations have been found between good patient experiences and clinical benefits (i.e. health outcomes, adherence to medications, and increased self-management) [4–7] and with healthcare cost reductions in areas such as reduced readmissions and length of stay [8–10].

This acknowledgment of patient experience as a key quality indicator of healthcare is reflected in the growing literature on the efficacy of interventions. Studies on systematic collection of patient experience surveys [11–14], reported trends suggesting improvement; however, there was no clarity on what contributed to the improvements in the patient experience.

### Interventions targeting patient experience

To date, interventions implemented at the point of care, targeting communication with patients, patient engagement in care processes and discharge planning were found to be associated with better patient experience outcomes. This suggests that interventions at the point of care, with a direct link to how patients experience the care delivery, could be more effective, as patient experience measures were designed and focused on what happens during the health care delivery interaction.

Interventions targeting attitudes of health professionals, patient/carer involvement and clinician-patient communication, at the point of care delivery were found to have the most significant impact on patient experience [15]. This was further supported by a qualitative study exploring patients’ perspectives where communication with and between patients and staff, interpersonal relationships with staff; engagement in care and discharge planning and the hospital environment determined a positive or negative patient experience [16]. The acknowledgement of the importance of clinician-patient communication and interpersonal skills has spurred the body of evidence in these areas. Studies in general communication training and patient education [17, 18] found that they are positively associated with the level of the patient experience. In contrast, a study on specific communication training for clinical consultations [19] found increased skill acquisition for health professionals, but the effect on patient experience was not rigorously evaluated. This suggests that while the relationship between these interventions and the patient experience was established, their efficacy and mechanism of action in different contexts are unclear.

### Patient experience

Patient experience measures are designed to find out what actually happens during the health care interaction, whether specific patient-centred care behaviours are carried out. These measures are collected through quantitative and qualitative approaches. Quantitative data may be collected through ward or hospital or national surveys. Qualitative data from interviews, focus groups, written and or video recording of patient stories on websites and observation and shadowing of patients. Each of these approaches has its strengths and limitations, and it is suggested that the triangulation of qualitative and quantitative studies is needed for a comprehensive understanding of patient experience [20, 21]..

Despite their limitations, patient experience surveys are preferred where the collection of standardised data from large representative patient populations for benchmarking is needed [22]. For example, the Picker Patient Experience Questionnaire [23] and the Hospital Consumer Assessment of Healthcare Providers and Systems (HCAHPS) used in the U.S. included questions about communication with nurses and doctors, interaction with other hospital staff (i.e. allied health, ancillary staff), the hospital environment, pain management, patient education, discharge process, overall experience and patient demographic characteristics.

These have been implemented in many countries. For example, in the UK, the National Health Service (NHS) introduced a national reporting system for surveys of patient experiences and reports publicly on service-level data [10, 24]. In the United States (US), standardised, national data on patient experience has been collected and reported publicly since 2011. In their efforts to improve patient care, Canada, Denmark, and the Netherlands have also established systems for collecting and publishing patient experiences measures [25–27].

### Aims of the study

With public reporting and the recognition of patient experience as a health service’s performance measure, health service providers are increasingly focused on improving patient experience. However, the evidence on interventions was limited by the lack of well-designed evaluation studies beyond single sites [28].

This study aimed to address this gap by evaluating the interventions used by public health services in Victoria to improve the experience of their patients, as indicated in outcome measures. First, to evaluate the target areas of the interventions and their individual and combined effects on the overall patient experience outcome measure. Second, the effects of the interventions on specific outcome measures of staff-patient communications, discharge planning, respect and dignity for patients, emotional support for patients, education of treatment/tests and the physical environment.

## Methods

### Design

This study is a secondary analysis of the Victorian Healthcare Experience Survey (VHES) [29] data for 2018, after the introduction of new reporting guidelines for improving patient experience in 2017. Surveys reported in September 2016 and September 2018 and the publicly available annual Victorian Quality Account [30] for 2017 from public health services in Victoria, Australia. Data from the VHES were provided to the investigators following completion of a standard application to Safer Care Victoria and the Victorian Agency for Health Information.

### Setting & study population

Victoria is the second most populous state in Australia with a population of 6.6 million people, most of whom (4.9 million people) live in the metropolitan and greater Melbourne area. Australia has a two-tier health system of public and private health services, where the funding and service delivery is shared between the Australian Federal government and the State governments. The Victorian state government is responsible for funding and managing public hospitals, regulating and licensing private hospitals, amongst other primary health and preventive services [31].

This study targeted public health services in Victoria that met the following criteria: (i) they had Victorian Healthcare Experience Survey (VHES) data for inpatients aged 18 years and above reported in September 2016 and September 2018 and (ii) had publicly available annual Victorian Quality Account for 2017.

### Sources of data

#### Victorian quality account

The Victorian Quality Account is a mandatory annual report where public health services are required to provide a qualitative description of the systems, processes and interventions used to fulfil various safety and quality indicators [32]. The individual health service reports were published on the Department of Health and Human Services (DHHS) Victoria website and individual health services’ websites. From 2017, with additional focus on patient experience, health services were required to report and describe the interventions taken to improve patient experience scores collected by the VHES [32]. As such, the Victorian Quality Account serves as a high quality source of data on interventions used by health services.

#### Victorian healthcare experience survey

The Victorian Healthcare Experience Survey (VHES) is a state-wide survey of patients’ public healthcare experiences required by the Department of Health and Human Services (DHHS) Victoria and is based on the internationally recognised work of the Picker Institute [29]. At the time of this study, surveys were administered to 10 patient categories: adult inpatients, adult emergency, maternity patients, paediatric inpatients, paediatric emergency, adult specialist clinics, paediatric specialist clinics, community health services, planned and emergency ambulance service and palliative care clients. The surveys were conducted monthly with adult inpatients, adult emergency and maternity patients and annually for the remaining seven patient categories [33].

In this study, the adult inpatient VHES surveys of 2016 and 2018 were selected as they included all health conditions. The adult inpatient VHES survey had a total of 92 questions.

### Data management

#### Qualitative description of interventions

The Victorian Quality Account 2018 reported details of interventions undertaken by the health service to address patient experience scores (measured by VHES). The Victoria Quality Accounts 2018 for the 59 health services were searched and downloaded from their respective official websites and cross-checked with the copy on the DHHS website. The dedicated section of the Victorian Quality Account, which focused on interventions for improving patient experience was extracted and analysed for each health service.

Documents, programs and policies and periodic reports were analysed using a five-step process; access, check validity, comprehend, analyse and apply the information in the form of extracted themes [34]. Content analysis was conducted using a deductive approach. Themes that emerged were assigned into the following intervention categories; patient-staff communication, staff-staff communication, respect and dignity, emotional support, integration of care, care continuity, discharge planning, treatment and disease education, waiting time and access and physical environment. These intervention categories were adapted from a taxonomy of professional interventions by the Cochrane Effective Practice and Organisation of Care (EPOC) Review Group [35] and a previous study [28] (see Additional file 1 for details).

Cross-case synthesis [36] was performed aggregating the themes that emerged into categories.

Before coding commenced, intervention categories and definitions were discussed and clarified. The excerpts from the Victorian Quality Accounts were coded for the presence and absence of the themes. The Kappa index was calculated for 10% of the health services (randomly selected) to determine the inter-rater reliability. In these, the extracted sections were coded by two coders independently. After the inter-rater reliability comparison, discrepancies were resolved by discussion and the remaining health services’ Quality Account reports were coded independently by one coder. All content analysis and coding of interventions from the Victorian Quality Account reports were completed before analysis of the VHES dataset to reduce a potential source of bias in coding if there was prior knowledge of any differences or trends in the VHES from 2016 and 2018.

The next step was to include and apply the categorical data of interventions to the quantitative data from VHES. This frequency distribution lists the number of occurrences for each category of intervention used by each of the 59 health services was created and added to the VHES dataset as additional intervention variables.

#### Quantitative data for outcome measures

The data for this study were pooled from raw unweighted responses from patients who were discharged from the services during the periods of April–June 2016 and April–June 2018, published in September 2016 and September 2018, respectively.

The VHES used a combination of three, four and five-point Likert response scale across the questions in the survey. Before data analysis, to standardise the response scale, the VHES survey responses were transformed to a universal 0–100 scale [37] with higher scores indicating a more positive response.

The relevant VHES survey questions were grouped into seven outcome measures, assessing the performance in the key patient experience areas of overall patient experience, communication, respect and dignity, emotional support, discharge planning, treatment and disease education and physical environment (see Additional file 2 for details).

#### Overall patient experience measure

The overall patient experience consisted of only one question in the VHES survey and enquired ‘Overall, how would you rate the care you received while in hospital?’ This measure was also used by the DHHS of Victoria as part of their performance monitoring and with the target set at 95% for public health services [38].

#### Communication experience measure

This measure consisted of six VHES questions regarding communication between staff and patient to assess the quality of communication experience.

#### Respect, dignity and emotional support measures

These measures assessed the level of respect and dignity (4 questions) and emotional support (5 questions) reported on the VHES questions. The questions categorised under these measures include those about respect, emotional support, the decision about care and privacy.

#### Discharge planning measure

The experience of care management in preparation for discharge was assessed by this measure, which consisted of four VHES questions. This measure was also used by the DHHS Victoria as part of their performance monitoring and with the target set at 75% for public health services [38].

#### Treatment and disease education measure

VHES questions (4 questions) about information provided to patients about their treatments, tests and operations were categorised into this measure.

#### Physical environment measure

This measure assessed the experience of the physical environment, VHES questions (2 questions) related to the cleanliness of hospital and ward environments were included.

### Statistical analysis

Descriptive analyses and univariate tests of between-group differences were conducted on the responses from the two VHES surveys (2016 and 2018). Confirmatory factor analysis on the VHES items that made up the outcome measures across the response groups from 2016 and 2018 [39] was conducted to test measurement invariance across the two cohorts groups. This analysis was conducted to examine whether respondents from both years interpret the VHES in equivalent ways [40, 41]. Measure invariance follows a sequence of steps. The first step is to establish configural equivalence. This is a baseline measure establishing that the same latent variables are being mapped in the two samples and that each latent variable has the same indicators. The second step is to constrain the regression weights of the indicators to be correspondingly equal across the 2016 and 2018 samples. This model is compared to the baseline to assess the degree of model worsening as a result of assuming equality of regression weights. If worsening is not significant, then metric equivalence is supported. The next step is to constrain the intercepts to equality (strong equivalence). This is then compared to the metric equivalence model. If the model statistics do not significantly worsen strong equivalence is supported, and any additional constraints will not invalidate the conclusion that there is measure equivalence/invariance.

The internal consistency of the outcome measures was established through the Cronbach alpha. The relationships among the variables are investigated using correlation coefficients and regression coefficients.

The association between each of the intervention categories (use and number of interventions) reported in 2017, and overall patient experience measure and other outcome measures in 2018 were examined using t-tests for dichotomous measures and one-way ANOVA for interventions with three or more levels with data from 2018. Finally, multiple regression was conducted to assess the contribution of the combination of related interventions to the overall patient experience measure. The data were compiled and analysed using IBM SPSS V.26 and AMOS V.26.

### Ethics approval

Ethics approval for this study was provided by the Monash University Human Research Ethics Committee (Project ID 20530).

## Results

### Sample characteristics

Data were contributed by 7709 respondents from the 2016 survey and 7497 respondents from 2018 survey (total *n* = 15,206). The mean ages of the 2016 and 2018 survey respondents were 64.3 years (SD =17.1) and 65.9 years (SD =16.5), respectively. The age in both 2016 and 2018 surveys had moderately negatively (left) skewed distributions. Details of the mode of hospital stay and category of health services are shown in Table 1. In both years, the metropolitan health services form the majority of the data.

**Table 1:** Mode of Hospital Admission and Category of Health Services

Characteristics	2016	2018
	*n (%)*	*n (%)*
***Hospital stays***		
Planned in advance	3994 (51.8)	3978 (53.1)
Emergency	2887 (37.4)	2717 (36.2)
Other	473 (6.1)	470 (6.3)
Missing	355 (4.6)	332 (4.4)
***Categories of Health Services***		
Metro	3119 (40.5)	3071 (41.0)
Regional	1965 (25.5)	1866 (24.9)
Rural	2270 (29.4)	2228 (29.7)
Missing	355 (4.6)	332 (4.4)

### Trends in patients experience scores between 2016 and 2018

There were 56 questions from the VHES with Likert type response scale; they were standardised, and mean scores were calculated for the overall patient experience measure and specific outcome measures. Internal consistency was checked for each set of questions, only those with Cronbach’s alpha (≥ 0.7), were used for further analysis as specific outcome measures. No valid set of questions for staff-staff communication, integration of care, waiting time and access, and care continuity were found and as such outcome measures in these areas were not included in the analysis (see Additional file 2 for list of questions).

There were strong positive correlations between the specific outcome measures of discharge planning, patient-staff communication, respect and dignity, emotional support and treatment and disease education outcome measures with the exception of the physical environment. (see Table 2).

**Table 2:** Correlations of Specific Outcome Measures

Outcome Measures	Cronbach’s α (No. of Qs)	1	2	3	4	5	6
1. Discharge Planning	0.8 (4)	1					
2. Physical Environment	0.8 (2)	.534	1				
3. Patient-Staff Communication	0.8 (6)	.847	.569	1			
4. Respect & Dignity	0.7 (4)	.769	.647	.913	1		
5. Emotional support	0.8 (5)	.820	.604	.983	.988	1	
6. Treatment & Disease Education	0.7 (4)	.781	.474	.863	.720	.805	1

The overall patient experience measure for 2016 responses (M = 93.4, SD = 13.1) and 2018 responses (M = 93.6, SD =12.9) was consistent between the 2 years. Despite no difference in the overall patient experience measure between 2016 and 2018 responses, the patient-staff communication measure and respect & dignity measure has increased mean scores indicating an improvement in those areas. On further examination nine out of 56 VHES questions also had increased mean scores, indicating an improvement in those areas. Notably, the same five questions had the lowest scores in 2016 and 2018, suggesting potential areas such as involvement in discharge planning and communication with doctors and caregivers to focus on for future interventions (See Table 3 for details).

**Table 3:** Mean scores on VHES questions in 2016 and 2018 responses

Outcome measures with significant differences between 2018 and 2016	Mean Score (2018) (2016)		***t***
Overall patient experience	93.60	93.36	1.12
Discharge planning	80.77	79.95	1.68^+^
Physical environment	93.72	93.45	1.32
Patient-Staff communication	87.75	87.01	2.07*
Respect & dignity	94.20	93.59	2.93***
Emotional support	89.45	88.53	1.76^+^
Treatment & disease education	86.26	86.13	0.22
**VHES questions with significant differences between 2016 and 2018**	**Mean Score (2018) (2016)**		***t***
How would you rate the politeness and courtesy of admissions staff?	95.44	94.90	3.09^**^
Did you feel friends and family were welcome to visit you?	97.26	96.50	2.91^**^
Were the nurses treating you compassionately?	94.76	93.96	2.86^**^
Were the doctors treating you compassionately?	92.95	92.18	2.32^*^
If you needed to talk to a nurse, did you get the opportunity to do so?	89.54	88.46	2.79^**^
How would you rate how well the doctors and nurses worked together?	86.97	86.27	2.65^**^
Did you see hospital staff wash their hands, use hand gel to clean their hands, or put on clean gloves before examining you?	90.80	89.54	3.01^**^
Do you think the time you had to wait from arrival until you were taken to your room or ward was?	88.35	87.22	2.52^*^
At other times during your hospital stay did you have enough privacy?	90.52	89.57	2.47^*^
**VHES questions with lowest scores**	**Mean Score (2018) (2016)**		***t***
If you needed to talk to a doctor, did you get the opportunity to do so?	82.22	81.43	1.44
How would you rate the hospital food?	79.60	79.28	0.88
Did you feel you were involved in decisions about your discharge from hospital?	77.61	76.23	2.30^*^
How much information about your condition or treatment was given to your family, carer or someone close to you?	69.08	70.01	1.15
Did you receive copies of communications sent between hospital doctors and your GP?	66.90	66.03	1.42

* = *p* < .05, two-tailed.** = *p* < .01, two-tailed.*** = *p* < .00, two-tailed. ^+^ = *p* < .05, one-tailed

Statically significant differences at *p* < .01 in the overall experience measure were found in comparing metropolitan, regional and rural health services in both 2016 and 2018 responses. Overall patient experience mean score of rural health services were higher than those of the regional and metropolitan health services for both years. However, the difference in mean score and effect size (eta squared = .03) was small. The statistical difference between the categories of gender identification (Male, Female) was found only in the 2018 responses, with a small magnitude of differences in the means (eta squared = .003). As such, further analysis based on these group differences were not conducted. See Table 4 for full details.

**Table 4:** One-Way ANOVA of Category of Health Services and Gender Identification on Overall Patient Experience

One-Way ANOVA					
	**(A) Metropolitan** **M (SE)**	**(B) Regional** **M (SE)**	**(C) Rural** **M (SE)**	**F-ratio**	**Differences**
2016	91.10 (.261)	93.45 (.297)	96.41 (.193)	114.03^***^	C > B > A
2018	91.37 (.262)	93.60 (.293)	96.67 (.197)	114.45^***^	C > B > A
**T-Test**	**(A) Male** **M (SE)**	**(B) Female** **M (SE)**		***t***	**Differences**
2016	93.60 (.232)	93.18 (.210)		1.33	–
2018	94.39 (.218)	92.93 (.219)		4.67^***^	A > B

* = *p* < .05, ** = *p* < .01, *** = *p* < .001

### Frequency of interventions reported by health services in 2017

**Fig. 1:**
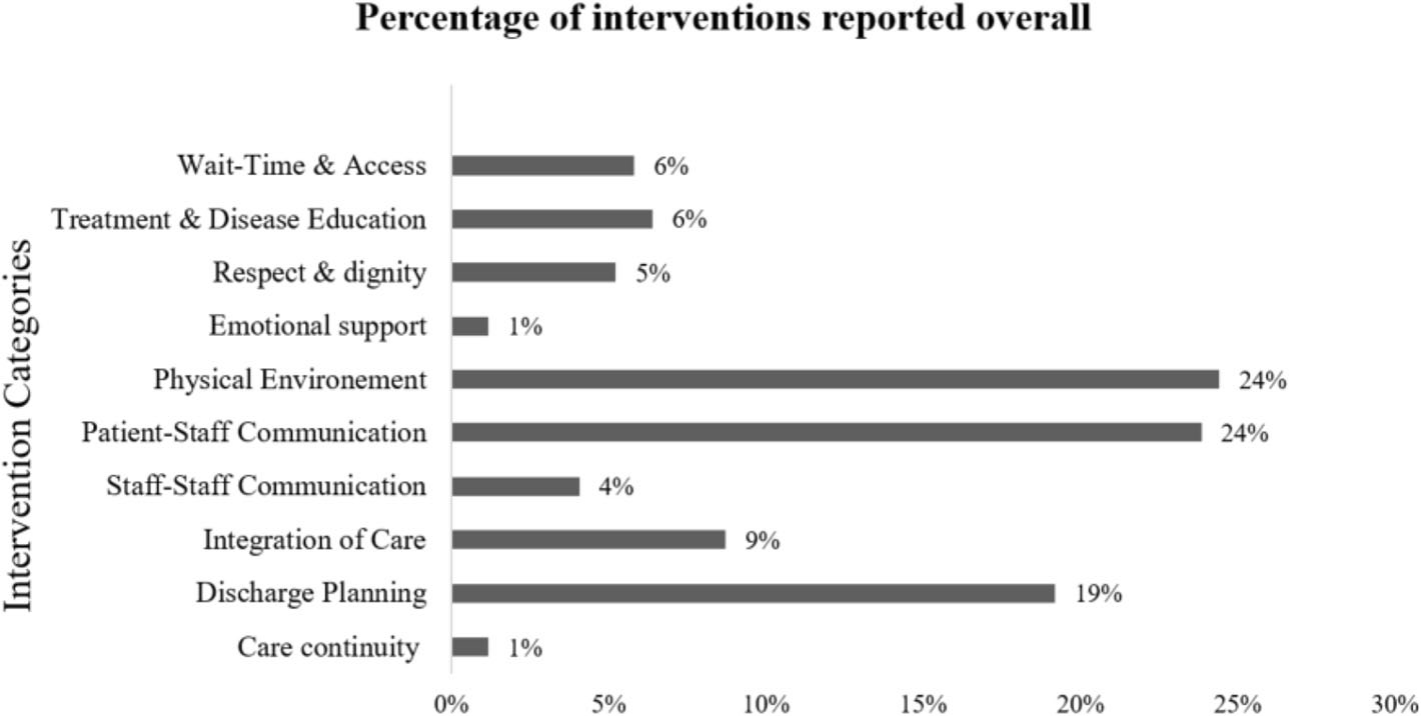
Interventions to Improve Patient Experience Measure

There were between one to four interventions coded for each of the health services, with reliability using Kappa index (*K* = 0.8). As depicted in Fig. 1, intervention categories most frequently reported were physical environment, followed by patient-staff communication and discharge planning. It is noted that patient-staff communication outcome measure were one of the measures with an increased in mean scores between 2016 and 2018, suggesting an improvement in this area.

### Measure invariance of 2016 and 2018 cohort

Testing for measurement invariance ensures that the comparison across the 2016 and 2018 cohorts were both meaningful and valid. The baseline model had *x*^2^ (154) = 2962.00; *p* < 0.001; *x*^2^; df ratio of 19.33; the RMSEA = 0.04 and the NFI, CFI and TLI all above 0.9 (see Table 5). Having established the baseline, testing for weak factorial invariance was conducted by constraining factor loading matrices. The result of *Δx*^2^ (*Δdf*) is 4.26; *p* < 0.005 for Model 1 showed some improvement from the baseline model, and the RMSEA improved with NFI, TLI, CFI consistent. The next step was conducted on testing for strong equivalence by adding additional constraints on Model 2 where the elements of the intercept matrices were constrained. The result of *Δx*^2^ (*Δdf*) is 1.74; *p* < 0.051 showed Model 2 improved in fit when assuming intercepts are equal. Further testing for strict factorial invariance by constraining the errors terms was conducted. The result of *Δx*^2^ (*Δdf*) is 6.30; *p* < 0.001 showed the Model 3 further improved when error terms are assumed equal. The testing for elegant factorial invariance showed that Model 4 with *Δx*^2^ (*Δdf*) is 3.83; *p* < 0.001 is a good fit with RMSEA, NFI, TLI, CFI were consistent with Model 3. The finding supports that the respondents from 2016 and 2018 interpreted the VHES survey measure in a conceptually similar way.

**Table 5:** Measure Invariance of 2016 and 2018 respondents

Model Comparison	***x***^**2**^ ***(df) p***	***Δx***^**2**^(***Δdf***) ***p***	***Δx***^**2**^ ***Δdf***	RMSEA	NFI	TLI	CFI
1. Baseline (configural invariance)	2962.00 (154) *p <* 0.001	19.33-	–	0.04	0.92	0.91	0.92
2. Model 1vs. Baseline Testing Invariance of $\hat{\varLambda}$ (weak factorial invariance)	3008.96 (165) *P <* 0.001	46.96 (11) *p* < 0.005	4.26	0.03	0.92	0.92	0.92
3. Model 2 vs. Model 1 Testing Invariance of $\hat{\tau}$ (strong factorial invariance)	3030.57 (178) *P <* 0.001	21.61 (13) *p* > 0.051	1.74	0.03	0.92	0.92	0.92
4. Model 3 vs. Model 2 Testing Invariance of $\hat{\theta}$ (strict factorial invariance	3113.59 (191) *P <* 0.001	82.02 (13) *p* < 0.001	6.30	0.03	0.92	0.93	0.92
5. Model 4 vs. Model 3 Testing invariance of $\hat{\psi}$ (elegant factorial invariance)	3117.42 (192) *p* = 0.00	3.83 (1) *p* < 0.001	3.83	0.03	0.92	0.93	0.92

### Association between interventions and VHES

The impact of these interventions was assessed firstly on the overall patient experience measure and subsequently on outcome measures derived from measuring specific aspects of care: 1) communication, 2) respect and dignity, 3) emotional support, 4) discharge planning, 5) treatment and disease education and 6) physical environment measure.

#### Impact of individual intervention category on overall patient experience measure

The differences on the overall patient experience measure associated with the number of interventions applied are shown in Table 6. There were significant differences with the application of integration of care interventions (*p* < .001) Two interventions were significantly better than one intervention which in turn was better than no intervention in improving overall patient experience. Care integration often involves changing existing clinical practices and processes across teams, and the result suggests that effective outcomes may require several coordinated interventions. There was no significant difference as a result of implementing discharge planning interventions.

**Table 6:** One-Way ANOVA and T-Test of Numbers of Interventions on Overall Patient Experience

**One-Way ANOVA**							
**Intervention category**	**(A) None** **M (SE)**	**(B) One intervention** **M (SE)**	**(C) Two interventions** **M (SE)**	**(D) Three interventions** **M (SE)**	**(E) Four interventions** **M (SE)**	**F-ratio**	**Differences (Post-hoc)**
Integration of care	93.26 (.167)	95.16 (.411)	96.51 (.550)			15.55^***^	C > B > A
Discharge plan	93.57 (.208)	93.62 (.233)	93.74 (.677)			0.38	–
Wait-time & Access	93.85 (.165)	92.80 (.373)		90.65 (1.38)		7.61^***^	A > B
Staff – Staff Communication	93.31 (.163)	96.92 (.362)	93.66 (1.02)			21.16^***^	B > A&C
Patient-Staff Communication	93.21 (.224)	95.02 (.238)	92.82 (.361)	89.50 (1.36)		16.23^***^	B > A > C&D
Physical environment	93.57 (.205)	93.20 (.282)	95.18 (.417)	91.51 (.865)	97.71 (.680)	7.71^***^	C > A&B&D E > A&B&C&D
**T-Test**							
**Intervention category**	**(A) None** **M (SE)**	**(B) One intervention** **M (SE)**				***t***	**Differences**
Care Continuity	93.69 (.155)	91.98 (.708)				6.36^*^	A > B
Respect & Dignity	93.20 (.165)	96.83 (.309)				57.17^***^	B > A
Emotional Support	93.95 (.156)	90.59 (.551)				45.91^***^	A > B
Treatment & disease education	93.51 (.153)	98.51 (.503)				19.71^***^	B > A

*p < .05, **p < .01, ***p < .001

Application of one intervention for staff-staff communication and patient-staff communication showed significant differences (*p* < .001) being higher scores than no intervention and two interventions. Similarly, for respect and dignity and treatment and disease education, significantly higher scores were found with one intervention compared to none. This suggests a carefully targeted intervention in staff-staff communication, patient-staff communication, respect and dignity and treatment and disease education could lead to a significant increase in overall patient experience score.

Overall patient experience measure did not increase with the use of waiting time and access to service, care continuity and emotional support interventions; no intervention was significantly higher than one or multiple interventions. This suggests that applying ineffective interventions in these areas decreased the overall patient experience.

### Impact of related interventions on overall patient experience measure

The effect of categories of intervention on the overall measure of patient experience is shown in Table 7. A hierarchical regression was used. Care continuity was significant (*p* < .01), but discharge planning was not significant. The two categories explained a negligible variance (R^2^ = .001). Model tested the effects of the next category of communication. This explained a negligible increased in variance (R^2^ = .003). Staff-staff communication was significant (*p* < .001); patient-staff communication was not significant. The next model on respect and dignity and emotional support significantly explained more variance in overall patient experience (R^2^ = .013). Respect and dignity was significant (*p* < .001), and finally, emotional support was negative and significant. This suggests emotional support interventions in this study reduced overall patient experience within this group category. Perhaps this indicates that emotional support interventions required further exploration of their acceptability and adherence by the healthcare professionals and patients. Model 4, with all the categories tested, explained slightly more variance in overall patient experience (R^2^ = .017). This suggests the need for prioritisation of intervention categories for their effect on overall patient experience when these categories of interventions were to be implemented.

**Table 7:** Regression Analysis Summary for Related Categories of Interventions on Overall Patient Experience

Grouped Interventions	Model 1		Model 2		Model 3		Model 4	
	*B*	*t*	*B*	*t*	*B*	*t*	*B*	*t*
Care Continuity	−.030	−2.55^**^					−.039	−3.21^***^
Discharge Plan	.005	.460					−.004	−.330
Staff-Staff Communication			.057	4.85^***^			.041	3.41^***^
Staff-Patient Communication			−.011	−.96			.024	1.79
Respect & Dignity					.080	6.81^***^	.085	7.06^***^
Emotional Support					−.069	−5.93^***^	−.077	−5.65^***^
R^2^	.001		.003		.013		.017	
*Δ* R^2^			.002		.010		.004	

* = *p* < .05, ** = *p* < .01, *** = *p* < .001

#### Impact of intervention on specific outcome measures

The impact of interventions (both application and level of application) on specific outcome measures were presented in Table 8. There were significant differences in staff-patient communication outcome measure (*p* < .001), where one intervention was significantly better than no intervention or multiple interventions. This suggests that focusing on one effective uniform communication intervention would provide a good experience for patients’ interaction with staff rather than using multiple interventions. Similarly, for respect and dignity and treatment and disease education significantly higher scores in the corresponding outcome measures were found with one intervention compared to none. Conversely, emotional support, no intervention was significantly higher than one or multiple interventions, suggesting that the intervention had a negative effect. Regardless of the number of discharge planning interventions, they made no significant difference. However, the mean scores (88–89%) of the discharge planning outcome measure across the number of interventions were all above the 75% target level set by the DHHS. The mixed findings on the physical environment could be due to the limited two questions (focusing only on cleanliness) in the outcome measure and not an accurate indicator of the changes in the physical environment. In summary, these results on specific outcome measures, are aligned with their impact on the overall patient experience measure.

**Table 8:** Impact of Interventions (both application and level of application) on Corresponding Outcome Measures

Interventions	**(A) None** M (SE)	**(B) One level** M (SE)	**(C) Two levels** M (SE)	**(D) Three levels** M (SE)	**(E) Four levels** M (SE)	F-ratio	Differences (Post-hoc)
Discharge Planning	89.40 (.326)	89.21 (.381)	88.68 (1.08)			.22	–
Physical environment	94.14 (.189)	92.28 (.283)	95.97 (.404)	92.47 (.686)	97.77 (.707)	17.82^***^	E > D&B&A
Staff-Patient communication	87.27 (.377)	89.35 (.447)	87.26 (.557)	82.88 (1.77)		7.49^***^	B > A&C&D A > D
Respect & dignity	93.89 (.162)	96.72 (.323)				36.41^***^	B > A
Emotional support	89.86 (.386)	86.21 (1.21)				9.78^**^	A > B
Treatment & disease education	86.18 (.421)	97.43 (1.52)				5.07^*^	B > A

* = *p* < .05, ** = *p* < .01, *** = *p* < .001

#### Relationship between overall patient experience measure and specific outcome measure

The correlations between the specific outcome measures and overall patient experience scores for each year (2016 and 2018) separately are presented in Table 9. The outcome measures and overall patient experience (*p* < .01) were highly correlated for both years and the correlations have generally increased in 2018 except for the discharge planning measure. This shows that the interventions were having a beneficial effect that is not reflected by the mean score in overall patient experience. This suggest one or other aspects of the experience could affect the overall experience, this finding is supported by findings from other studies [42, 43].

**Table 9:** Correlations of overall patient experience and specific outcome measures

		2018						
		**Overall patient experience**	**Discharge planning**	**Physical environment**	**Staff-Patient communication**	**Respect & dignity**	**Emotional support**	**Treatment & disease education**
**2016**	**Overall patient experience**		.477**	.491**	.719**	.609**	.753**	.544**
	**Discharge planning**	.483^**^		.355**	.583**	.425**	.509**	.466**
	**Physical environment**	.458^**^	.314^**^		.472**	.475**	.483**	.317**
	**Patient-staff communication**	.692^**^	.578^**^	.462^**^		.645**	.819**	.680**
	**Respect & dignity**	.598**	.423**	.446**	.641**		.721**	.491**
	**Emotional support**	.748^**^	.520^**^	.447^**^	.796^**^	.705^**^		.626**
	**Treatment & disease education**	.536^**^	.509^**^	.329^**^	.700^**^	.481^**^	.638^**^	

** = *p* < .01

## Discussion

This study found that the level of overall patient experience of the 59 health services in 2016 and 2018 was consistent (over 93%) but below the set target (95%). A modest increase in mean scores in nine questions from 2016 to 2018 was also found. The interventions reported were not found to change the overall patient experience scores significantly. This could suggest that the interventions contributed to the maintenance of the scores. However, to break through the plateau of the overall patient experience score, further evaluation of the interventions’ efficacy and implementation is needed.

Nevertheless, in examining the categories and effect of interventions reported by health services in 2017, differences were found on the overall patient experience and specific outcome measures in the 2018 VHES scores.

The Victorian Government and DHHS had set out a state-wide design, service and infrastructure plan for the health services from 2017 to 2037 [44]. This could explain why the highest number of interventions reported by health services were in the area of improvement to the physical environment.

The next categories most frequently chosen and reported by the 59 health services were communication between staff and patients and discharge planning. Some examples of communication interventions included the introduction of communication aids such as whiteboards for staff to invite patients to ask and answer their questions, ‘teach-back’ method asking patients to explain in their own words the information given [45] and a campaign to remind staff to introduce themselves and their names to patients. The discharge planning interventions included the introduction of discharge planning discussion at the bedside with patients and reminders for staff to include and involve carers and families in discharge planning meetings. It was noted that respect and dignity and emotional support interventions were used least. It could suggest a lack of efficacious interventions in these areas or knowledge of them.

The results of this study provide a working basis for prioritising interventions for health services and policymakers. Firstly, by the categories or focus areas of interventions; next, the ‘dosage’ or number of interventions required to have an effect on overall patient experience.

When discharge planning and staff-patient communication interventions are already implemented and associated with a consistent level of overall patient experience (as in the case of this study), prioritising the focus areas of staff-staff communication and respect and dignity interventions are more likely to increase overall patient experience. However, the use of care continuity and emotional support interventions was associated with a decrease in the level of the patient experience. It is possible that the interventions were not evidence-based, fit for the context or not well implemented. Further examination of the efficacy and choice of interventions in these areas is needed. It is also possible that certain interventions require an extended implementation period, and their effect may not be evident in a year.

The next finding is about the optimal ‘dosage’ for interventions. When examining the ‘dosage’ and its effect on the overall patient experience or corresponding outcome measure, a similar pattern was observed. In targeting communication amongst staff and between staff and patients, one intervention is associated with more positive patient experience than multiple interventions. This was also found in the areas of respect and dignity and treatment and disease education where only single intervention was implemented and was effective. Speculatively, one well-designed and evidence-based intervention that was implemented uniformly in a health service would have a more significant impact than multiple interventions implemented in different departments. This study contributes to the body of literature on the efficacy of single versus multiple interventions [46, 47], where the evidence on the efficacy is caveat by the context and nature of the intervention. Nevertheless, in the context of the Victorian public inpatient services, using one evidence-based intervention in the areas at the point of care mentioned above is associated with improvement in overall patient experience.

### Strengths and limitations

To the knowledge of the authors, this is the first study to examine and integrate the routinely collected qualitative and quantitative data on improvement efforts on patient experience in Victoria, Australia. This study identifies state-wide trends on areas and the impact of interventions on patient experience with system-level implications beyond an evaluation of intervention in pilot sites.

Another contribution is in expanding the use of the VHES instrument beyond the one question overall patient experience measure and the discharge planning measure that were used by policymakers as performance indicators. This study explored the potential of extracting other specific outcome measures from VHES to evaluate the different aspects of the patient experience. However, a review of the sensitivity of the measure could not be conducted using just the results of the survey in this study alone.

There are a few limitations to this study; the majority of the interventions focused on changing staff behaviours at the point of care and broader organisational-level interventions were not examined and not in the scope of the study. Despite the large sample, the data were only at two points in time (2016 and 2018) and not able to measure changes or interventions that may take longer to take effect, future studies could include data from more than two years of survey to have a more indicative trend. Besides, the findings are not causal and provide no direct explanations of the mechanism behind these associations. We also cannot rule out the possibility of reporting bias since the interventions are based on self-report from the health services.

### Implications for health services

For health service practitioners designing and planning future interventions for improving patient experience, there could be significant benefits to consider the areas of respect and dignity and emotional support, least targeted in this study, as they are highly correlated and account for most of the effect on overall patient experience. They were also identified in evidence-based practices and recommendations [16, 48] to improve patient-centred care and patient experience.

Another important consideration is the selection and design of the intervention needed in the relevant context. This could be achieved through identifying the target behaviour for change, targeting barriers faced by the staff for their practice change and using a theory-informed intervention [49] where the mechanism of action is identified and can be tested and replicated. This may require significant time and resources to do in healthcare settings. As shown in this study, prioritising and focusing on implementing one intervention for each area instead of multiple interventions is more likely to improve outcomes and optimise resources utilisation.

### Implications for policy

These findings confirm the impact of policy levers such as guidelines and funding-related performance targets on health services’ quality improvement activities in patient experience. However, there are other opportunities for policymakers to support the improvement of the patient experience. Firstly, support the pilot and evaluation of evidence-based interventions in the areas of respect and dignity and emotional support to break through the plateau of patient experience scores. Secondly, encourage the appropriate ‘dosage’ of interventions by engaging and supporting large-scale evidence-based of a single intervention at any one time in the areas of communication, respect and dignity and treatment and disease education.

### Research implications

There are still many unanswered questions about the rationale and mapping of interventions to patient experience improvement areas. Future research is needed to identify the specified behaviours to change, examine the context and barriers before mapping and choosing the interventions required. This will contribute to the body of evidence to inform the implementation of more evidence-based interventions.

## Conclusion

In conclusion, this study contributes to the evidence in exploring the impact of interventions on patient experience among inpatient service users in Victoria, Australia. Despite no significant change in overall patient experience, it is encouraging that health services were maintaining a consistent level of the patient experience. It is the hope that the findings will encourage further support in identifying and implementing evidence-based interventions as part of routine practice. In the interim, these findings demonstrate that when the patient experience is measured as an essential aspect of health services performance, it leads to efforts at continuous improvement and recognition of the importance of patients’ views.

## Supplementary Information

**Additional file 1:.** Codebook of categories and description of interventions

**Additional file 2:.** VHES questions mapped to outcome measures

## Data Availability

The data generated or analysed during this study available from corresponding author on reasonable request.

## Abbreviations

DHHS: Department of Health and Human Services
EPOC: Effective Practice and Organisation of Care
HCAHPS: Hospital Consumer Assessment of Healthcare Providers and Systems
NHS: National Health Service
VHES: Victorian Healthcare Experience Survey
