# Supplementary material for: Victorian healthcare experience survey 2016–2018; evaluation of interventions to improve the patient experience
Source: BMC Health Serv Res. 2021 Apr 7;21:316. doi: 10.1186/s12913-021-06336-0 (PMC8028773; doi:10.1186/s12913-021-06336-0)
Supplement: Supplementary file 1 — Additional file 1:. Codebook of categories and description of interventions [file 12913_2021_6336_MOESM1_ESM.docx]

Additional file 1

Victorian Healthcare Experience Survey 2016 – 2018; evaluation of interventions to improve the patient experience.

Eunice Wong^1,4^, Felix Mavondo^2^, Lidia Horvat^3^, Louise McKinlay^3^, Jane Fisher^4^

^1^ BehaviourWorks Australia, Monash Sustainable Development Institute, Monash University, Melbourne, Australia

^2^ Department of Marketing, Monash University, Melbourne, Australia

^3^ Safer Care Victoria, Department of Health and Human Services Victoria, Australia

^4^ School of Public Health and Preventive Medicine, Monash University, Melbourne, Australia

Corresponding author: Eunice Wong, email: [eunice.wong@monash.edu](mailto:eunice.wong@monash.edu) postal address: BehaviourWorks Australia, Monash Sustainable Development Institute, PO Box 8000, Monash University LPO, Clayton, VIC 3800, Australia.

**Additional file 1: Codebook of categories and description of interventions**

|  | | | |
| --- | --- | --- | --- |
| Sources of reference in the development of coding of interventions: | | | |
| 1. Existing taxonomy from Effective Practice and Organisation of Care (EPOC). EPOC taxonomy | | | |
| 2. Wong, E., Mavondo, F., & Fisher, J. (2020). Patient feedback to improve quality of patient-centred care in public hospitals: a systematic review of the evidence. BMC health services research, 20(1), 530. https://doi.org/10.1186/s12913-020-05383-3 | | | |
|  | | | |
| Interventions in Communication and Responsiveness to patient categories were not found in the EPOC taxonomy, additional definitions were derived from Wong, Mavondo and Fisher (2020). | | | |
| Communication: Communications with patients and their carers or between staff members | | | |
| Responsiveness to patient: The level of professionalism, competence and support provided by healthcare staff. | | | |
| Categories of interventions adopted (adopted from the EPOC taxonomy* and from systematic review on interventions for quality improvement from patient feedback**) for coding are in the coding descriptions | | | |
|  | | | |
|  | Intervention categories (* = EPOC, **= Wong et. al 2020) | Description | |
| 1 | Professional practice* | Role expansion or task shifting* | Expanding tasks undertaken by a cadre of health workers or shifting tasks from one cadre to another, to include tasks not previously part of their scope of practice. |
|  |  | Length of consultation* | Changes in the length of consultations |
|  |  | Staffing models* | Interventions to achieve an appropriate level and mix of staff, recruitment and retention of staff, and transitioning of healthcare workers from one environment to another, for example interventions to increase the proportion of healthcare workers in underserved areas. |
|  |  | Care pathways* | Aim to link evidence to practice for specific health conditions and local arrangements for delivering care. |
|  |  | Case management* | Introduction, modification or removal of strategies to improve the coordination and continuity of delivery of services i.e. improving the management of one “case” (patient) |
|  |  | Communication between providers* | Systems or strategies for improving the communication between health care providers, for example systems to improve immunization coverage in LMIC |
|  |  | Comprehensive assessment* | A multidimensional interdisciplinary diagnostic process focused on determining a person’s medical, psychological and functional capability to ensure that problems are identified, quantified and managed appropriately |
|  |  | Continuity of care* | Interventions to reduce fragmented care and undesirable consequences of fragmented care, for example by ensuring the responsibility of care is passed from one facility to another so the patient perceives their needs and circumstances are known to the provider. |
|  |  | Discharge planning* | An individualized plan of discharge to facilitate the transfer of a patient from hospital to a post-discharge setting. |
|  |  | Disease management* | Programs designed to manage or prevent a chronic condition using a systematic approach to care and potentially employing multiple ways of influencing patients, providers or the process of care |
|  |  | Integration* | Consolidating the provision of different healthcare services to one (or simply fewer) facilities. |
|  |  | Packages of care* | Introduction, modification, or removal of packages of services designed to be implemented together for a particular diagnosis/disease, e.g. tuberculosis management guidelines, newborn care protocols. |
|  |  | Referral systems* | Systems for managing referrals of patients between health care providers |
| 2 | The physical environment** | Physical ** | Physical environment, i.e. replacing chairs, layout, adding and changing the physical space |
|  |  |  | Sensory environment, i.e. reduction in noise, introducing music, quiet spaces |
|  |  |  | Hygeine |
|  |  |  | Food |
|  |  |  | Sanitation, i.e. clean toilets, beds |
|  |  |  | Amenities - access to wifi |
|  |  |  | Changes in change physical spaces, i.e change from inpatient to outpatient clinic, ED to specialist clinic for appointment |
|  |  | Access** | Waiting time |
|  |  |  | Clinical access |
|  |  |  | Treatment access. E.g. contact to make appt, admission,etc |
|  |  |  | Acess to diagnosis |
|  |  |  | Delay in admissions |
|  |  |  | Failure/delay in visit |
| 3 | Communication** | Staff -staff dialogue** | Systems for information sharing, i.e IT system, patient charts, handover |
|  |  |  | Complete and accurate information |
|  |  |  | Sharing information, i.e. patient concerns, queries, test results |
|  |  | Patient-staff dialogue** | Doctor patient-relationship, i.e. introducing themselves, calling patient by name |
|  |  |  | Listening to patient and families |
|  |  |  | Interpersonal communication |
|  |  |  | Explanation to patients |
|  |  |  | Agree on care expectations i.e clarify on the care and what to expect from the care process and outcomes |
|  |  |  | Invite participation |
|  |  |  | Decision sharing |
| 4 | Responsiveness to patient** | Respect and dignity** | Show concern |
|  |  |  | Respect for wishes |
|  |  |  | Respond to needs |
|  |  |  | Sensitive |
|  |  |  | Ask preferences |
|  |  |  | Dignified service |
|  |  | Emotional support** | Emotional support |
|  |  |  | Acknowledgement |
|  |  |  | Empathy |
|  |  |  | Caring |
|  |  | Professionalism** | Attitude of staff |
|  |  |  | Competent behaviour |
|  |  |  | Staff commitment |
| 5 | Patient Education** | Patient rights** | Rights i.e. Healthcare charter |
|  |  | Treatment & Disease education** | Treatment options |
|  |  |  | Disease education |
|  |  |  | Care navigation: i.e. information at point of presentation |
