# Supplementary material for: Victorian healthcare experience survey 2016–2018; evaluation of interventions to improve the patient experience
Source: BMC Health Serv Res. 2021 Apr 7;21:316. doi: 10.1186/s12913-021-06336-0 (PMC8028773; doi:10.1186/s12913-021-06336-0)
Supplement: Supplementary file 2 — Additional file 2:. VHES questions mapped to outcome measures [file 12913_2021_6336_MOESM2_ESM.docx]

Additional file 2

Victorian Healthcare Experience Survey 2016 – 2018; evaluation of interventions to improve the patient experience.

Eunice Wong^1,4^, Felix Mavondo^2^, Lidia Horvat^3^, Louise McKinlay^3^, Jane Fisher^4^

^1^ BehaviourWorks Australia, Monash Sustainable Development Institute, Monash University, Melbourne, Australia

^2^ Department of Marketing, Monash University, Melbourne, Australia

^3^ Safer Care Victoria, Department of Health and Human Services Victoria, Australia

^4^ School of Public Health and Preventive Medicine, Monash University, Melbourne, Australia

Corresponding author: Eunice Wong, email: [eunice.wong@monash.edu](mailto:eunice.wong@monash.edu) postal address: BehaviourWorks Australia, Monash Sustainable Development Institute, PO Box 8000, Monash University LPO, Clayton, VIC 3800, Australia.

**Additional file 2: VHES questions mapped to relevant specific outcome measures**

| **Outcome measures** |
| --- |
| **Overall patient experience measure (n=1)** |
| Q76 Overall, how would you rate the care you received while in hospital? |
|  |
| **Communication (Staff-Patient) (n=6)** |
| Q21 If you needed to talk to a doctor, did you get the opportunity to do so? |
| Q27 If you needed to talk to a nurse, did you get the opportunity to do so? |
| Q32 Did the staff treating and examining you introduce themselves and their role? |
| Q33 How often did the doctors, nurses and other healthcare professionals caring for you explain things in a way you could understand? |
| Q43 If you had any worries or fears about your condition or treatment, did a health professional discuss them with you? |
| Q77 Do you feel that you were listened to and understood by the people looking after you in hospital? |
|  |
| **Respect and Dignity (n=4)** |
| Q9 How would you rate the politeness and courtesy of admissions staff? |
| Q45 Were you given enough privacy when being examined or treated? |
| Q46 At other times during your hospital stay did you have enough privacy? |
| Q78 Overall, did you feel you were treated with respect and dignity while you were in hospital? |
|  |
| **Emotion Support (n=5)** |
| Q2 How would you rate the politeness and courtesy of staff in the ED? |
| Q22 Were the doctors treating you compassionate? |
| Q28 Were the nurses treating you compassionate? |
| Q44 Do you feel you received enough emotional support from hospital staff during your stay? |
| Q47 Do you think the hospital staff did everything they could to help manage your pain? |
|  |
| **Discharge Planning (n=3)** |
| Q63 Were you given enough notice about when you were going to be discharged? |
| Q64 Did you feel you were involved in decisions about your discharge from hospital? |
| Q65 On the day you left hospital, was your discharge delayed for any reason? |
|  |
| **Treatment and disease education (n=4)** |
| Q38 How much information about your condition and treatment was given to you? |
| Q51 Did you receive sufficient information about any medication you were given while in hospital (e.g. purpose, side effects and how to administer the medication)? |
| Q53 Did a member of staff explain why you needed these test(s) in a way you could understand? |
| Q54 Did a member of hospital staff explain the results of the tests in a way you could understand? |
|  |
| **Physical environment measure (n=3)** |
| Q12 In your opinion, how clean was the hospital room or ward that you were in? |
| Q13 How clean were the toilets and bathrooms that you used in hospital? |
| Q35 Did you see hospital staff wash their hands, use hand gel to clean their hands, or put on clean gloves before examining you? |
